# Empowering Through Psychodrama: A Qualitative Study at Domestic Violence Shelters

**DOI:** 10.3389/fpsyg.2021.600335

**Published:** 2021-03-31

**Authors:** Yiftach Ron, Liat Yanai

**Affiliations:** ^1^Graduate School of Creative Arts Therapies, Kibbutzim College, Tel Aviv, Israel; ^2^Hebrew University of Jerusalem, Jerusalem, Israel

**Keywords:** psychodrama, domestic violence, women's shelters, victimhood, empowerment, action research

## Abstract

Psychodrama is a therapeutic method in which the stage is used to enact and reenact life events with the aim of instilling, among other positive changes, hope and empowerment in a wide range of populations suffering from psychological duress. The therapeutic process in psychodrama moves away from the classic treatment of the individual in isolation to treatment of the individual in the context of a group. In domestic violence situations, in which abusive men seek to socially isolate their victims from family and friends, the social support that psychodrama provides can positively influence the psychological health and well-being of the participants. This qualitative study examines the manner in which psychodramatic treatment can empower abused women residing in domestic violence shelters and help them regain control of their lives. An action research study of domestic violence survivors living in a women's shelter in Israel, over a 12-month period, demonstrates the role of psychodrama therapy in promoting the reduction of anxiety, stress, guilt, and self-blame, while reinforcing perceptions of self-worth and confidence. These findings contribute to our understanding of the potential of psychodrama in helping reshape life roles and reframe experiences within a creative process, with the aim of facilitating a transition from powerlessness to powerfulness among vulnerable populations.

## Introduction

Domestic violence is described as a pattern of abusive behaviors, including physical, sexual, and psychological forms of maltreatment, that are carried out to exert and demonstrate power over the victim; it is primarily perpetrated by men on their female partners (Walker, 1996; Meloy and Miller, 2011; Lombard and McMillan, 2013). The global problem of violence against women crosses all religions and cultures. At the UN's fourth International Conference on Women in 1994, every participating country reported that domestic violence was occurring within its borders (Chen, 1995; Paxton and Hughes, 2014). Global estimates published by the World Health Organization in 2012 indicate that 30% of women have experienced physical violence by their intimate partner (World Health Organization, 2012; Rollè et al., 2019). Furthermore, it is important to note that domestic violence is a largely hidden crime, and most of the cases are unreported (Davis et al., 2003; Gracia, 2004; Dillon et al., 2013). In the United States, it has been estimated that a woman is abused by an intimate partner every 9 s, and roughly 8.7 million women are physically abused by a male partner each year (Roberts, 2005). In Israel, according to Wizo's (2020) violence index, over 200,000 women (about 7–8% of adult women in Israel) are subjected to domestic violence. The global incidence of domestic violence and its extremely detrimental effects require an immediate call for action, but women who are abused in their own homes often feel helpless (Roberts, 1998). Domestic violence can rob them of their confidence and self-esteem, stripping them of the courage needed to leave their abusive situation (Chen, 1995; Choice and Lamke, 1997; Metz et al., 2019).

One of the most common governmental interventions to protect women and children has been the opening of domestic violence shelters (Sommerfeld and Shechory Bitton, 2016). In 1985, the United States passed the Domestic Violence Prevention Act, which authorized the funding of state domestic violence organizations, agencies, and shelters. The purpose of such shelters includes both immediate and long-term goals: (1) help abused women find safety, (2) empower women regain control of their lives, (3) and provide therapeutic interventions to deal with the ramifications of abuse (Dutton, 2006).

Before abused women make the decision to seek help and enter a domestic violence shelter, many are in denial of their situation, blame themselves for the abuse, and see no alternative possibilities (Filson et al., 2010; Eckstein, 2011; Sommerfeld and Shechory Bitton, 2016). Most women suffer abuse for years before gaining the courage to leave and seek help, and even then the effects of victimization do not automatically disappear once the women have left their abusive homes (Griffing et al., 2002; Anderson and Saunders, 2003). A study conducted in South Africa reveals a focus on victimhood and powerlessness in the narratives of women's shelter residents (Schalkwyk et al., 2014). In recent years, literature on domestic violence is moving from an emphasis on women's powerlessness and victimhood toward a focus on empowerment (Schalkwyk et al., 2014; Bucuta et al., 2018).

### Therapeutic Interventions With Abused Women

When a woman escapes her abuser and moves to a shelter, her most immediate need is for safety. Yet, she also faces challenges such as having to search for a new home, find work, filing legal action, and dealing with the psychological harms caused by the abuse. All of these factors put women at high risk of returning to their abusive partners. In fact, many do return to their abusers after having resided in a shelter for a period of time (Griffing et al., 2002; Anderson, 2003; Metz et al., 2019). The research literature suggests several explanations for why they do so, including: lack of economic resources and social support, effects of early childhood trauma, and feelings of disempowerment (Choice and Lamke, 1997; Metz et al., 2019).

Therefore, once immediate safety needs are met, most shelters offer several modalities of treatment and therapy designed to change thought patterns and allow for the development of new behaviors (Jonker et al., 2015; Sommerfeld and Shechory Bitton, 2016). These psychological interventions address three major dimensions: (1) *cognitive*: self-esteem, thought processing, perceptions of others, and perceptions of the world at large; (2) *psychological*: anxiety, anger, and depression; and (3) *interpersonal*: relationships and difficulties with trust and intimacy (Dutton, 2006). A comparative study in Israeli women's shelters of residents' perceptions of their abusive marital relationships revealed two pervasive themes in their thought processing: dominance and submission (Sommerfeld and Shechory Bitton, 2016). There was an imbalance of power between themselves and their spouses; many described their abusive partners as hostile and domineering and wished that they themselves would become more independent and powerful in the future. This suggests that therapies in shelters should address the concepts of independence, power, and submissiveness and try to empower residents to cultivate a sense of control over their lives (Shostack, 2001; Sommerfeld and Shechory Bitton, 2016).

Yet, in traditional therapy there is a structural power imbalance between the therapist and the patient that could potentially perpetuate feelings of powerlessness in abused women (Carlson, 1997). This is in addition to some other limitations of individual therapy such as the lack of group support or less of an opportunity to learn about others with similar issues and how they address their problems (Malan, 1979; Sharf et al., 2010). Group psychotherapy, in contrast, provides the participants with an experience of equality of status with the other participants and even with the therapist (Dreikurs, 1955). Unlike one-on-one therapy, in which there is only a single relationship between two people, the group is based on multiple relationships: member to group, member to member, group to leader, and leader to group (Riva et al., 2004; Stone, 2016). The interpersonal dimension—a group member's sense of acceptance and belonging; a personal allegiance and commitment to the group; and the trust, support, and compatibility felt among the group—plays a significant role in the therapeutic process that occurs in group settings (Wise and Nash, 2019). Rudolf Dreikurs emphasized the dimension of equality that exists in group therapy, in which individuals are valued for who they are in the group and for their self-disclosure and honesty, not for what they have achieved in their lives (Dreikurs, 1955; Fehr, 2003). Other studies have found that individuals who felt understood and protected in group therapy reported greater improvement in overall well-being (MacKenzie and Tschuschke, 1993; McDermut et al., 2006).

Yalom describes the change that takes place in group therapy as a complex interplay of interpersonal experiences that he defines as therapeutic factors. He presents 11 primary factors of the group therapeutic experience: instillation of hope, universality, imparting information, altruism, corrective recapitulation of the primary family group, development of socializing techniques, imitative behaviors, interpersonal learning, group cohesiveness, catharsis, and existential factors (Yalom, 1995). Yalom's description of *universality* as a therapeutic factor that helps group members realize that they are not alone in their suffering and the problems they face corresponds with Foulkes's concept of *mirror reaction* (Fehr, 2003). Foulkes, the founder of the group analysis approach, described a liberating and relieving process of reflection, in which an individual in the group see him/herself reflected in one of the other group members. He emphasized the common interest that exists within the group, and viewed group therapy as a space for the unconscious to become conscious (Foulkes, 1964; Fehr, 2003). According to Rogers, who coined the term encounter group, the group adds a dimension of humanity to interpersonal relationships, and is of great value in a society that encourages the suppression of emotions (Rogers, 1970; Rutan et al., 2014).

A particularly important feature of group therapy with abused women is the social support offered by the group. In many cases abusive men sought to socially isolate their partners from family and friends (Browne, 1987; Theran et al., 2006). Therefore, social support networks are an essential factor in helping abused women recover from violent relationships (Tan et al., 1995). A study by Tan et al. (1995) examined the relationship between social support variables, psychological well-being, and experience of further abuse. Results showed a strong relationship between social support and the psychological well-being of abused women.

### Empowering With Psychodrama

The unique nature of psychodrama group therapy is beneficial in ways that traditional psychotherapy is often inadequate. The psychodrama group acts as an accommodating space for coping with experience of distress of the participants by creating a space for self-expression and a human encounter, mutual support, and sharing (Ron, 2018). Roine and others describe the ability of psychodrama to evoke spontaneity and uncover creativity in difficult patients (Holmes and Karp, 1991; Roine, 1997; Blatner, 2000; Schacht, 2007). Farmer (1995) highlights the way in which the psychodramatic stage allows participants to approach their feelings and thoughts in situations where the verbal dialogue of analytic psychotherapy is limited. These techniques are especially beneficial for vulnerable populations, such as at-risk adolescents, alcoholics, drug addicts, and those coping with anorexia (Karp, 1994; Karatas, 2011; Orkibi et al., 2014, 2017a,b; Orkibi and Feniger-Schaal, 2019).

A recent study on the use of psychodrama with abused women shows how psychodramatic methods can support the women in their recovery process and facilitate changes in their victim role (Bucuta et al., 2018). Abused women typically engage in various coping strategies that are related to the way they perceive the abusive relationships in which they are trapped. One of these coping strategies is characterized by guilt and self-blame (Filson et al., 2010; Sommerfeld and Shechory Bitton, 2016). These feelings are often provoked by the abuser's complaints regarding the woman's performance in her roles as a wife and mother (Miller and Porter, 1983; Valor-Segura et al., 2011). Psychodrama in general, and the technique of role reversal in particular, can allow abused women to reshape their perceptions on their own life roles (Yaniv, 2012; Bucuta et al., 2018). In role reversal, the protagonist exchanges roles with another person represented by an auxiliary ego, and together they enact a significant interpersonal situation; doing so allows the protagonist to view their world from the viewpoint of the other and to explore the behaviors and feelings that are embedded in the roles that they tend to play (Moreno et al., 1955; Holmes and Karp, 1991; Kellermann, 1994). Participants are encouraged to reexamine life choices and expand their role repertoire by developing new roles, both within the self and also in relation to how they interact with others. While much of role reversal reflects human relationships, the protagonist may choose to present and reverse roles with parts of themselves that they need to understand better or confront (Kellermann, 1994). Thus, role reversal and role playing can facilitate both the learning of new coping skills and a shift in perceptions of the other and the self (Dayton, 1994).

Another technique that allows participants the opportunity to see themselves and the world through another's perspective is the doubling technique. Doubling is the attempt, made by the psychodramatist or by an auxiliary group member, to express the unvoiced thoughts or feelings of the protagonist, enabling them to gain clarity and express a deeper level of emotion (Blatner, 1996; Moreno et al., 2000). The double affords the protagonist a sense of visibility and facilitates expression of thoughts and feelings (Blatner, 2000; Fox, 2008). This process helps them to develop additional self-knowledge and more sense and meaning of the situation (Holmes and Karp, 1991; Dayton, 2005). In addition, the auxiliary group member, who has succeeded, through their capacity for empathetic projection in feeling their way into the inner world of the protagonist, can feel valued and heard. This occurs regardless of the accuracy of the doubling, because even if the protagonist rejects or corrects the interpretation, it has given the auxiliary a voice and the protagonist the opportunity to clarify their feelings (Ron, 2018).

The current study is intended to serve as a framework for understanding the benefits of psychodrama group therapy in addressing mindsets and behavior patterns related to victimhood and powerlessness, and in helping abused women residing in domestic violence shelters regain control of their lives. This study seeks to contribute to the understanding of the processes that take place within the setting of psychodrama group therapy in a women's shelter, and of the potential of psychodrama in helping reshape life roles and reframe experiences within a creative process, with the aim of facilitating a transition from powerlessness to powerfulness among survivors of domestic abuse.

## Methods

### Action Research

This study was conducted using an action research (AR) approach that combines methodical investigation with practical action in the societal or communal setting. The concept of AR originates with the work of Lewin (1946) and John Collier (Deshler and Ewert, 1995) on the subject of intergroup dynamics, and is associated with activities designed to produce social change, liberate oppressed groups, and create an egalitarian society. Today, the term AR is used in a broader sense, to describe various kinds of research carried out by practitioners (McIntyre, 2008; Mertler, 2011). This investigational approach, which in the past was commonly found mainly in education and teaching research, is now an accepted method in various fields such as social work, organizational behavior, healthcare, public health, mental health, and others (Meyer, 2000; Koshy et al., 2011; Hutchinson and Lovell, 2013).

AR sees the practitioner-researcher as the primary research tool. The research is characteristically descriptive, drawing its data from the natural framework of the field work, emphasizing the process itself, and attaching importance to the subjects' own interpretation of the observations (Stringer, 1999; Greenwood and Levin, 2007).

Using the AR approach this study followed a psychodrama therapy group over a 12-month period in a women's shelter in Israel.

### The Study Setting and Participants

The women's shelter is located in the center of Israel. It can hold up to 12 women, who are each assigned daily responsibilities, including cooking meals, cleaning rooms, and doing maintenance work around the facility. Some of the women work outside of the shelter, but their hours are restricted and must align with the nightly curfew. Their children attend kindergartens and schools in the community. The shelter includes a nursery that provides day care for children ranging from 2 to 4 years of age.

There is no time limit on how long a resident can remain in the facility. Because there is no specific duration of services, the psychodrama group was an open group, allowing for turnover and variability of its participants (Turner, 2011; Miller and Mason, 2012); there were between 6 and 10 participants in each session. Group members' ages ranged from 20 to 65.

The sessions took place once a week, lasting about an hour. Each session began with introductions and a “group pulse-check” in which the participants shared with the group how they were. This was followed by an active warm-up, then enactment of a psychodramatic vignette, and group sharing as a closure. The therapeutic goal was to provide a safe and protected space for self-expression, reflection, and interpersonal encounter, to help the participants practice new ways of interacting and communicating and to allow them to voice their thoughts and feelings in order to advance a process of healing and empowerment among the participants.

The group was led by two female psychodrama trainees, and the contents of each session were processed in both individual and group supervision meetings that took place on a weekly basis.

### Materials and Data Analysis

The study utilized participant observation. Materials include detailed verbatim transcripts of all the therapy sessions during the course of the study, as well as other documents including drawings and letters written by the participants during the group sessions and collected by the therapists. Materials created by the participants during the group sessions were collected in order to keep them for the participants and were returned to them at the end of the process. These materials were also analyzed as part of the supervision process to help the facilitators gain a better understanding of the participants and their needs. Although the findings of the study are illustrated through examples taken from the verbatim transcripts, it is important to note that all the documents collected were carefully analyzed as part of the process of revealing the main thematic categories which are presented in the following section.

The verbatim transcripts were written immediately following each session by the group therapists and processed in weekly supervision sessions. All materials were used with the express consent of the group participants and with the institution's approval. Materials were edited and any identifying information was removed.

Our analysis is based on the *grounded theory* approach which emphasizes the generation of hypotheses and concepts based on data derived from the research (Strauss and Corbin, 1998). In line with this approach, several stages of qualitative content analysis (QCA) were undertaken (Berg, 2004; Forman and Damschroder, 2007; Vaismoradi and Snelgrove, 2019). The first phase included a thematic analysis of each document. In written documents, units of analysis were paragraphs or segments of text from the transcript. At the same time the entire text was also treated as a single segment. The intention was to enable the necessary dismantling of each session into specific units of content while retaining the ability to see them in their original context (Berg, 2004).

The initial analysis revealed numerous thematic categories emerging from each transcript. After rereading a given transcript, the number of categories was reduced by combining similar categories and focusing on those that emerged most relevant. Next, the transcripts were integrated based on the categories that they had in common. These categories were scrutinized again for centrality (repeated appearances across different sessions) for the connections between them, and for their relevance to theory and to the study's topic (Berg, 2004; Roth, 2005).

## Findings

The findings of the study are illustrated through examples taken from the verbatim transcripts and presented in two main thematic categories: (a) the role of psychodramatic techniques such as role reversal and the doubling technique in empowering the participants, and (b) empowering through the sharing circle in the group. The quoted passages are excerpts from the verbatim transcripts written by the therapists immediately following the group sessions.

### Empowering Through the Use of Psychodramatic Techniques

One of the most prominent techniques in which frequent use was made during the therapeutic process is *role reversal*. Role reversal was used in the group to help the participants gain insight into the experience of others and to deepen their emotional experience in a way that would allow them to explore and reshape their life roles. The following example is from a psychodramatic vignette with M, a 29-year-old mother of two who immigrated to Israel from Russia with her husband and his mother. Having no family in Israel, M was dependent on her husband and endured years of physical and emotional abuse. A preschool teacher of her son, who noticed bruises on her face, encouraged M to leave her home and helped her to seek shelter for herself and her two children. During one of the sessions, M expressed her disappointment and frustration with her 9-year-old daughter:

*I wasn't born in Israel, my Hebrew is minimal, and I feel in many ways my daughter expects me to help her in ways that I simply cannot. Yesterday my daughter was doing her homework and was constantly asking me to come and help her. She knows that I don't know how to read and write Hebrew, and yet she keeps insisting that I come and sit with her. After 20 minutes I started screaming at her! Then my daughter started crying, threw her homework on the floor and left the room. I don't know what to do anymore*.

The therapist asked M if she would like to work as the protagonist and reenact the scene she just described. M agreed and chose an auxiliary ego to play her 9-year-old daughter.

M role playing as Daughter: Mom, can you come and help me with my homework. Mom, mom, can you come and help me!

(M role-reverses back and forth with her daughter)

*M: You need to do it yourself*.

Daughter: Mom, I need your help!

M: You know that I don't understand, so stop asking me!

Therapist: M, do you mind if I am your double for a moment as you respond to your daughter?

*M: Ok*.

*Therapist as the double for M: I understand that it is difficult for you. And I want to be there for you. It must be hard doing your homework all by yourself*.

*Therapist turns to M: Do you think this is something you can say to your daughter? If so, let's try role playing it again*.

*M to Daughter: I understand this is hard for you. But I don't speak the language. I don't understand. But if you want, I can still sit with you*.

*Daughter: You never sit with me. You always say you are busy*.

M: What is the point of sitting with you if I cannot help you!

*Therapist as the double for M: Deep down I am embarrassed that I cannot help you. So I pretend to act busy so that I do not have to face it*.

*M (with tears in her eyes). Yes. It is true, I am embarrassed*.

*M to daughter: I will try and be there for you. Even though I cannot help you with your homework, maybe all you need is to not feel alone*.

*Daughter: You never want to just play with me. You always have a list of things you need to do. I'm never on that list*.

In this vignette, M, by stepping into the role of her daughter, was able to realize that perhaps her daughter was just asking for her attention and not necessarily for concrete help. Instead of feeling frustrated and helpless because she was unable to help with her daughter's homework, M had the opportunity to feel valuable as a mother just by showing her daughter empathy and love.

As often happens in protagonist-centered psychodrama, through M's vignette, not only the protagonist but also other participants in the group could process similar difficulties they have been facing in their lives. Immediately after M's scene, S, an Israeli-born 34-year-old woman who came to the shelter with her 3-year-old daughter, responded to M's work, and shared her experience with the group:

*S: I also feel like I am not meeting the needs of my daughter. She has a temper tantrum over everything; the food she eats, she doesn't want to get dressed, doesn't want to shower, she just screams and screams and screams. And now I find myself screaming at her back*.

*Your story made me feel like I am not speaking the same language as my daughter and maybe I need to find a different way to communicate with her. Screaming isn't helping any of us*.

At one of the subsequent sessions, M shared with the group that one afternoon, while her daughter was doing her homework, M sat down next to her. She put her hand on her daughter and said, “How about afterward we take a break and make a chocolate cake together?”. The understanding that all her daughter needed was her presence and attention allowed M to let go of her insecurities as a mother and focus on the quality of time they spent together as mother and daughter.

Another powerful tool that has been used repeatedly in the group was the *doubling technique*. The double in psychodrama is meant to act as an additional “I,” which allows a protagonist to express and share thoughts and feelings that may be difficult to articulate into words, and pose repressed conflicts while providing a sense of safety and support. The double played a significant role in the therapeutic work with T, a 19-year-old woman of Ethiopian descent who came to the shelter with her 2-year-old daughter. T, although born in Israel, was isolated from the Israeli culture and brought up with a strict Ethiopian culture. Married from the age of 16, T lived next door to her husband's parents and was subjected to abuse and violence by her husband and her father-in-law. T's older sister, who had felt that T was living in terror and was unable to defend herself, helped her to find a shelter as she feared for her sister's life.

The first time T joined the group, she was extremely closed and shy and did not utter a word the whole meeting. As the weeks progressed, T slowly began opening up, but she still found it very difficult to share her feelings and opinions with the group. However, after some time, T chose to share with the group a dream she had the previous night. In her dream T is standing in a courtroom in front of her husband and father-in-law, who are screaming and throwing things at her. The judge asks her to sit on the stand and state her case. T seats down on the witness stand but she is frozen and cannot utter a word. Her husband and father-in law continue screaming at her until the judge finally throws T out of the courtroom.

T explained to the group that she was scheduled soon to meet her husband at an Israeli courthouse. The therapist asked her if she would like to work as the protagonist and try to prepare herself for the meeting. She agreed. The therapist asked T to set up the room as she would imagine the courtroom to look. T placed five chairs around the room. She said she would like to try to speak to her father-in law. T set up two chairs, one facing the other and chose a group member to play her father-in-law.

*Therapist: T, if you don't mind, before we start the scene, I would first like to ask you some questions*.

*T: Ok*.

Therapist: What are you doing at the courthouse?

*T: I am asking for a divorce*.

Therapist: Why are you crying?

*T: (long silence) I am afraid to see my father-in-law and husband*.

Therapist: What scares you?

*T: That they will scream at me and I will not be able to answer. In our religion, no matter how you are treated, one must respect their elders*.

Therapist: I must remind you that now we are on the psychodrama stage and you have the freedom to act however you feel, to say whatever you want. Do you want to try?

*T: Ok*.

Therapist: So we will go back to the scene in the courtroom. Please look at your father-in-law. What would you like to say?

*T: (Long silence). I don't know. All I can imagine is him screaming at me*.

Therapist: Let's try to role-reverse. Can you be your father-in-law for a moment?

*T sits in as her father-in law*.

Therapist to father-in-law: You know T cannot find the words to speak to you. Do you know why?

T as father-in-law: T has no right to speak. She is a disgrace to this family!

Therapist asks T to role-reverse back to herself and respond to her father-in-law. T doesn't say anything, she is looking down at the floor.

Therapist: T, may I be your double for a moment?

*T nods her head*.

*Therapist as the double for T: I don't mean to disrespect you, that is not my goal. But I do not believe I should be treated this way. I have the right to voice my opinion*.

*Long pause, T is crying*.

Therapist: T, do you agree with what I have said?

*T nods her head*.

*Therapist: Can you repeat it in your own words*.

*T shakes her head that she cannot*.

*Therapist as the double for T: I don't deserve to be treated this way.I have the right to voice my opinion. I never tried to hurt anyone. I only want to protect my children*.

*After a long pause, T finally looks up*.

T to her father-in-law: My children have no right to be treated this way

The vignette continued for quite some time. The use of the doubling technique allowed T for once to voice her opinion, even if only in a psychodramatic setting. This had an impact also on T's groupmates, as her work related to their own experiences and memories. One of them was D, a 57-year-old woman who suffered physical and emotional abuse for over 30 years. D left her husband and moved to the shelter when she knew her children had already grown up and no longer needed her protection. In the sharing phase that followed T's work, D shared her own experience with T and the group:

*D: Your scene reminded me of what scared me the most at home, and that was dinner time. My husband would come home and if I didn't have everything in the exact order, cooked the exact way he liked it, he would charge at me. Sometimes even when I tried to make everything perfect he would find one thing, one time the salt container was not fully filled. The way you described your feeling in the courtroom, that's how I felt at every meal…that fear of speaking up. But hearing your story I realize how ridiculous his anger was. I didn't deserve it. My flaws did not deserve such abuse*.

In one of the subsequent meetings, T shared with the group her experience at the courthouse:

It was a very difficult time. I felt my body sweating and still I could not look into the face of my husband. But when the judge asked me to speak, I was able to tell her what I wanted. I was able to ask for my freedom

In addition to role reversal and the double, other psychodramatic techniques were used in the group during the year. One of them is the *Magic shop*, a psychodramatic technique in which an imaginary shop is opened by the therapist or by a member of the group under the supervision of the therapist: what it sells are not goods, but instead the participants are given an opportunity to buy and sell different characteristics and emotional states. During this activity the “owner” and “customer” negotiate a deal. The task of the owner is to make a realistic deal with the customer (the protagonist) that can foster a commitment for change. Here is an example from one of the group sessions:

The therapist asked the group who would like to begin. L, a 22-year-old mother of two, raised her hand. L gave birth at the age of 18 to twin boys. Six months after the birth of her children, her partner lost his job and started to drink heavily. He became physically abusive which led L to leave him and seek shelter.

Therapist: Welcome to my magic shop. What are you interested in buying today?

*L: I want to sell my heart and buy a stronger one*.

Therapist: We cannot take your heart, that is something we do not buy here since it is uniquely yours. What we can offer you are things to make your own heart stronger. Can you think of anything that will make your heart stronger?

*L: Power and patience*.

Therapist: What kind of power do you want? Can you give me a statement of power?

*L: Yes.that I have the power and right to be respected*.

Therapist: Can you say it again, but in a louder voice?

*L: I have the power and right to be respected*.

Therapist: I am not convinced that if I sell you power and respect that you will really use it. Your tone of voice is not convincing. You say it almost in a whisper, as if you are embarrassed or ashamed. Do you really feel you deserve to be respected?

*L: (long silence).....Not always*.

Therapist: Why not?

*L: Because I uprooted my children and took them away from their friends, their community. I uprooted them to a place where they feel alone and scared*.

Therapist: Then why did you do it?

L: What do you mean?

Therapist: Why did you leave and seek shelter?

*L: Because my children were in danger. My husband is a dangerous person*.

Therapist: So even though things are difficult, do you believe you and your children are in a safer place now?

*L: Yes.Yes, we are safer*...

Therapist: Did your husband treat you with respect?

*L: No. No he did not*.

Therapist: And do you really want to buy the power to ask for respect at this magic shop?

*L: I do*.

*Therapist: Ok then, convince me. Ask me again*.

*L: (in a slightly louder voice). I have the power and right to be respected*.

Therapist: Yes, we have some power and respect left at the shop. Although it is a very popular item at our shop. What will you sell us in return?

*L: Guilt and shame*.

Therapist: And how much guilt and shame do you have to sell?

L: What do you mean?

Therapist: If you had to think of it in terms of kilos, how heavy is the load you are selling?

*L: (pauses for a long time)...I don't know, probably 200 kilo*.

Therapist: Wow. That is quite a heavy load. I will have to check that I have enough storage space...(pretending to check). Ok, it may be possible that I take this heavy load from you. But how can I be sure that you will not return and ask for it back?

*L: (silent and confused)*.

Therapist: If I sell you power and respect, are you sure that you will not want your shame and guilt back in the future?

L: I don't want it back!

Therapist: Are you sure?

L: Yes!

Therapist: Are you sure???

L: Yes!!!!

*Therapist: Done deal. (Therapist extends her hand out to L for a proper handshake)*.

Another example of the use of the magic shop technique is taken from a psychodramatic work with D who came to the shelter at the age of 57 after being subjected to abuse and violence by her husband for 30 years:

*D: I would like to buy courage*.

*Therapist: And what will you do with courage*.

*D: I will stay here*.

Therapist: Stay where?

*D: At the shelter. I will stay and not go back home*.

Therapist: Why would you go back home?

*D: My children. They don't understand how bad it was for me. They just keep pressuring me to go back home to their father*.

Therapist: And what will you sell in return for courage?

*D: Fear. I have so much fear*.

Therapist: Fear of what?

D: That I am not making the right choice. That maybe I should go back, for my children…May I ask to buy one more thing in addition to courage?

Therapist: Yes. What do you want?

*D: I want faith; faith and belief that I have done the right thing*.

Being a customer or owner in the magic shop can be a very powerful and empowering experience. Allowing participants to choose for themselves what they want to sell and buy puts them in charge of their choices, rather than their decisions being influenced by what a therapist may suggest she needs. At the same time the participants learn that they cannot get anything of value without giving something away. There is no growth, no transformation, without giving something up.

### Empowering Through the Sharing Circle in the Group

In addition to the use of role reversal, doubling and other psychodramatic techniques, the group itself acted as a space for self-expression and empathy, mutual support, relatedness, and sharing. The sharing phase in psychodrama is the phase in which group members share their personal life experiences as they relate to the work of the protagonist. In practice, the psychodrama activity in the women's group did not necessarily focus on one protagonist, and there was not always a clear separation between the main activity and the sharing phase. Within the group, the sharing circle was a space where participants could share their feelings, their troubles, and whatever else they were experiencing.

The women in the group empowered each other, as common themes and challenges emerged and served as the framework for a support system, helping alleviate a sense of loneliness within individual situations and struggles. This space of empathy, mutual support and sharing often evoked an experience of universality; a discovery that the individual is not alone in her experience and in her distress. Sometimes this happened right at the beginning of the group sessions:

*The psychodrama session opens with a group “pulse check”. The participants are asked to express their emotional state*.

Therapist: If you could express yourself as a season, how would you describe yourselves this morning?

*R: Winter. Even though I can see the sun shining through the window, it feels like winter*.

Therapist: What does winter feel like for you?

*R: It's cold. I feel like there's a storm inside me. Sometimes I think I would be happier if I went back home*.

*V: I felt the same way when I first came to the shelter. I think I felt worse than I felt living at home*.

*R: I miss my bed. I miss my kitchen. I miss my furniture*.

*Therapist: I want you to lift up your head and look around the room. Look at all the women here. R looks around the room with tears in her eyes*.

*T: It was also very hard for me in the beginning. I couldn't fall asleep for weeks. But after, when I realized I was surrounded by women who felt the same, it became easier*.

Here, R shared with the group her emotional state and the difficulties she has been coping with. This was followed by sharing of similar experiences by V and T. A dimension of universality was established within the group, where individuals could see themselves in one of the other group members. T aptly described it in her own words when she shared her experience with R and with the group: “*It was also very hard for me in the beginning… but after, when I realized I was surrounded by women who felt the same, it became easier*.”

When a woman shared a problem, a distressing experience, or a painful sentiment during the group sessions, the participants often used the final sharing circle to offer support, empower, and encourage one another:

*O, a 32-year-old mother of three was the protagonist. Her scene focused on her 8-year-old son, normally a shy boy, who suddenly began to misbehave at school. O believed leaving her home had a serious impact on her son and she was consumed with guilt. As the enactment ended, the women formed a circle to take part in the sharing*.

*S: I would like to begin. The first time I was at a shelter was three years ago. I had to pull my daughter out of her school and it was very difficult for her. She didn't want to leave her home. She would cry every morning, complaining how much she missed her friends. In the end, I left the shelter. I didn't give myself or my daughter enough time to adjust. I just went back… and now here we are again. But this time I realize that my daughter will adjust. Slowly she is making new friends*.

*T: I also struggle a lot with how my children feel. It's still very hard, but what choice do I have*.

*C: Sometimes I can't sleep at night because I have so much guilt. Guilt about leaving my home, leaving my close family*.

The following session, when asked how she felt being the protagonist a week earlier, O said that the women in the group continued to support her and that she felt understood and less alone. Here we can see how the group sharing has become a space of mutual support as S, T, and C tried to encourage O. This happened many times during the course of the group; the circle of sharing allowed the women to express their distress and feelings of guilt, regret, and anxiety, and at the same time to act as “therapeutic agents” for each other.

## Discussion

The lives of women who experience ongoing domestic violence and abuse are plagued with powerlessness and victimhood (Filson et al., 2010; Eckstein, 2011; Sommerfeld and Shechory Bitton, 2016). Although turning to a shelter requires courage and determination, and constitutes a first step toward breaking the cycle of abuse, the effects of victimization do not automatically disappear once the women have left their abusive homes (Griffing et al., 2002; Anderson and Saunders, 2003; Baholo et al., 2015). Therefore, therapeutic interventions in domestic violence shelters should address mindsets and behavior patterns related to victimhood, self-blame and powerlessness, and try to empower patients to cultivate a sense of control over their lives (Shostack, 2001; Sommerfeld and Shechory Bitton, 2016).

Psychodrama, as an action-based method, provides a rehearsal stage to practice new ways to behave in relationships (Moreno et al., 2000; Bucuta et al., 2018). It offers a safe environment to explore and examine life experiences that one has accepted as truth, and has the potential to facilitate action and change in patients' lives. The foundation of one's beliefs can be reexamined by reenacting particular events, thus potentially leading to a change in self-narratives and the belief system as a whole (White, 2007; Azoulay and Orkibi, 2015; ter Avest, 2017).

The findings of this study show the potential of psychodrama group therapy to allow a once voiceless victim the opportunity to release pent-up emotions that can foster new learning and behavior patterns (Dayton, 1994). Throughout the course of the psychodrama women's group, expressions of fear, and feelings of incompetence and powerlessness were frequently manifested in the group. Tackling these themes by using psychodramatic techniques—role reversal, the doubling technique, and others—accompanied with the support of the group and the participation in the sharing circle, produced movement from feelings of helplessness and despair to feelings of empowerment and control.

Role reversal in psychodrama can allow participants to view their world from the viewpoint of the other and to explore the behaviors and feelings that are embedded in the roles that they tend to play in their lives (Moreno et al., 1955; Holmes and Karp, 1991; Kellermann, 1994; Yaniv, 2012). This is of particular importance in therapeutic work with abused women, where feelings of guilt and self-blame are often provoked by the abuser's complaints regarding the woman's performance in her roles as a wife and mother (Miller and Porter, 1983; Valor-Segura et al., 2011). Role reversal was frequently used in the women's group to enable participants to see themselves through the eyes of others in a way that would allow them to expand their self-narrative and reshape their point of view on their own life roles (White, 2007; Yaniv, 2012; Bucuta et al., 2018). Use of role reversal had an additional empowering effect when women enacted their lives on the psychodramatic stage: through role reversal women were able to gain control over how antagonists were embodied in the scene. Reclaiming control, even if only in a psychodramatic setting, is an important part of the process of healing and empowerment of women who have gone through domestic abuse (Shostack, 2001; Dutton, 2006; Sommerfeld and Shechory Bitton, 2016).

Another tool that was used repeatedly in the group along with role reversal was the psychodramatic double. The double gave voice to women who struggled to express themselves, offered interpretation or insight while providing a sense of safety and support, and enabled expressions of identification and empathy among group members. This reflects the concepts of Moreno and his successors of the double as an “additional I,” which allows the protagonist to better sense that they are visible and facilitate expression of thoughts and feelings that may be difficult to formulate into words (Holmes and Karp, 1991; Blatner, 2000; Fox, 2008; Ron, 2018). In the women's group, the double played an important role in creating a special environment in which creativity and free expression of thoughts could blossom. Such a freeing environment is of great importance in breaking the chain of abuse and promoting the possibility of change and growth (Shostack, 2001; Filson et al., 2010).

In addition to the use of psychodramatic techniques, the study illustrates the role of sharing in the group as scaffolding for mutual support among the participants. In this space, the women could share their feelings and distress with the group and sense the attentiveness of other participants, who occasionally offered responses as well. The women in the group could return to this space to sense the *universality* of their experiences (Yalom, 1995), or the *mirror reaction* as termed by Foulkes (Fehr, 2003), in which participant discover that they are not alone in their distress; that their fellow group members cope with similar distress and they share it with the group (Fehr, 2003). This phenomenon corresponds with the concept of *common humanity* (seeing one's experiences as part of the larger human experience rather than as separating and isolating) in Neff's discussion of the construct of *self-compassion* (Neff, 2003). This is the quintessence of what Moreno described as the fabric of life and the human encounter that comprises the psychodrama group (Moreno, 1953; Blatner, 2000; Fox, 2008). In situations of domestic violence, in which women have been socially isolated by their abusers from family and friends (Lanier and Maume, 2009; Eckstein, 2011), the quality of social support provided by psychodrama group therapy can have a strong positive influence on the psychological health and well-being of participants (Tan et al., 1995).

### Limitations of the Study, Reflexivity, and Future Directions

The findings of this study may contribute to the understanding of the benefits of psychodrama in treating women in domestic violence shelters, but this study is not without limitations.

One methodological issue concerns the *action research* method and the danger of blurring boundaries between researcher and practitioner, and between research and clinical action. Nevertheless, AR offers significant advantages—its relevance as applied research, as well as the deep familiarity of the researcher with the participants. As regards the current study it is important to refer to our role and engagement as practitioner-researchers. LY was a trainee psychodramatist during the course of the study and one of the female facilitators of the women's group. YR is a male psychodramatist, trainer, and researcher. While LY's familiarity and deep connection with the participants were invaluable and allowed us a better understanding of subtle nuances of participants' behaviors and emotions, we feel that YR's more distant position had a balancing effect on our research. At the same time, we tried to be aware of, and restrain possible effects of the asymmetry of power related to seniority, academic status and gender position of each of us.

Secondly, it is important to note that this study is descriptive in nature and based on qualitative research methods that are not intended to provide a precise measurement of the effects of psychodrama therapy or participants' difficulties and needs, nor does it purport to definitively demonstrate a direct causal relationship between these two factors. Instead, this study relies on anecdotal data in which clinical judgement and interpretation play a major role. Alternative explanations are available to account for the processes and events described other than those provided here.

## Conclusion

This study contributes to our understanding of the benefits of psychodrama group therapy for residents of women's shelters in dealing with feelings of self-blame, helplessness, and lack of control. Previous studies point to mindsets and behavior patterns related to victimhood and powerlessness among survivors of domestic abuse and violence (Griffing et al., 2002; Anderson and Saunders, 2003; Filson et al., 2010; Eckstein, 2011; Schalkwyk et al., 2014; Sommerfeld and Shechory Bitton, 2016). Another strand of research describes the therapeutic benefit of psychodramatic techniques (Holmes and Karp, 1991; Dayton, 1994; Kellermann, 1994; Blatner, 1996, 2000; Moreno et al., 2000; Fox, 2008), and its effectiveness in treating particularly difficult populations for whom traditional psychotherapy's usefulness is limited (Karp, 1994; Vieira and Risques, 2007; Karatas, 2011; Orkibi et al., 2017b; Bucuta et al., 2018; Ron, 2018). The unique contribution of this study is the close encounter that it provides to practitioners and researchers with the processes that take place within the setting of psychodrama group therapy in a domestic violence shelter, and with the ways in which psychodrama can reduce manifestations of fear, self-blame, and powerlessness among survivors of domestic abuse.

## Data Availability Statement

The datasets presented in this article are not readily available because of reasons of confidentiality and participants' privacy. Requests to access the datasets should be directed to Liat Yanai, liatyanai@gmail.com.

## Ethics Statement

Ethical review and approval was not required for the study on human participants in accordance with the local legislation and institutional requirements. The patients/participants provided their written informed consent to participate in this study.

## Author Contributions

LY and YR collaborated on the design of the study, the analysis, and writing of the manuscript. LY facilitated the data collection and wrote the first draft of the manuscript. YR revised the preliminary drafts and supervised the research process. All authors contributed to the article and approved the submitted version.

## Conflict of Interest

The authors declare that the research was conducted in the absence of any commercial or financial relationships that could be construed as a potential conflict of interest.
